# MALT1 positively correlates with Th1 cells, Th17 cells, and their secreted cytokines and also relates to disease risk, severity, and prognosis of acute ischemic stroke

**DOI:** 10.1002/jcla.23903

**Published:** 2021-07-17

**Authors:** Xia Chen, Xuemei Zhang, Ling Lan, Guoyao Xu, Yanchun Li, Shaoming Huang

**Affiliations:** ^1^ Department of Anatomy Hunan University of Medicine Huaihua China; ^2^ Department of Anatomy Guangxi Medical University Nanning China; ^3^ Department of Neurology The First Affiliated Hospital of Hunan University of Medicine Huaihua China

**Keywords:** acute ischemic stroke, disease severity, MALT1, recurrence‐free survival, Th1 and Th17 cells

## Abstract

**Background:**

This study aimed to explore the association of mucosa‐associated lymphoid tissue lymphoma translocation protein 1 (MALT1) with acute ischemic stroke (AIS) risk and also to explore its association with T helper type 1 (Th1) cells, Th17 cells, disease severity, and prognosis in AIS patients.

**Methods:**

One hundred twenty first‐episode AIS patients and 120 non‐AIS patients with high‐stroke‐risk factors (as controls) were recruited. Besides, in the cluster of differentiation 4‐positive (CD4^+^) T cells, the MALT1 gene expression was detected by reverse transcription quantitative polymerase chain reaction; meanwhile, Th1 and Th17 were detected by flow cytometry. Moreover, serum interferon (IFN)‐γ and interleukin (IL)‐17 were determined by enzyme‐linked immunosorbent assay.

**Results:**

MALT1 expression was increased in AIS patients compared with controls and also it could differentiate AIS patients from controls, with an area under curve of 0.905 (95% confidence interval: 0.869–0.941). In AIS patients, MALT1 positively correlated with Th1 cells, Th17 cells, IFN‐γ, and IL‐17. Besides, MALT1 positively correlated with the National Institutes of Health Stroke Scale score. Furthermore, the Kaplan‐Meier curve and univariate Cox's regression analyses showed no correlation of MALT1 high expression with recurrence‐free survival (RFS) in AIS patients, although after adjustment using multivariant Cox's regression, high MALT1 expression independently correlated with worse RFS in AIS patients.

**Conclusion:**

MALT1 expression is increased and positively correlates with disease severity, Th1 cells, and Th17 cells, whose high expression severs as an independent risk factor for worse RFS in AIS patients.

## Graphical abstract

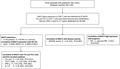

## 1 INTRODUCTION

Acute ischemic stroke (AIS) is a cerebrovascular disease characterized by vascular occlusion in the cranial blood vessels.^1^ Notably, AIS remains a huge concern in the modern society due to its high rates of comorbidity and mortality.^2^, ^3^ Regarding its management, endovascular intervention (including thrombectomy and thrombolysis) is commonly used in treating AIS patients.^2^, ^3^, ^4^ However, only a few AIS patients are in treatable stage owing to the difficulty of early detection and immediate medical attention, although majority of them miss the optimal therapeutic period, resulting in poor prognosis.^3^, ^5^ Recently, some novel biomarkers have been identified as prognostic factors in AIS patients (such as 25‐hydroxyvitamin D).^6^ Therefore, identifying potential and reliable factors to monitor disease timely identification and improve prognosis in AIS patients is necessary.

Mucosa‐associated lymphoid tissue lymphoma translocation protein 1 (MALT1) is a key mediator in the nuclear factor (NF)‐κB signaling pathway which is involved in lymphocyte activation, proliferation, and survival via its link to different receptors.^7^, ^8^, ^9^ Meanwhile, previous studies disclose that MALT1 regulates a cluster of differentiation 4‐positive (CD4^+^) T‐cell differentiation into T helper type 1 (Th1) cells and Th17 cells.^10^, ^11^, ^12^, ^13^ Furthermore, apart from that, MALT1 enhances vascular inflammation and endothelial barrier disruption in several studies.^14^, ^15^, ^16^, ^17^ Because MALT1 may enhance inflammatory cytokine production in endothelial cells and induce endothelial dysfunction, and these physiological changes indicate further atherosclerosis, thrombosis formation, and AIS occurrence and also MALT1 regulates Th cell differentiation and their related inflammatory cytokine excretion; therefore, we hypothesized that MALT1 measurement might play a pivotal role for AIS management.^10^, ^11^, ^12^, ^18^, ^19^, ^20^ In our preliminary study with a relatively small sample size, we discovered that MALT1 was increased in AIS patients compared with controls. Therefore, we conducted this study and aimed to investigate the correlation of MALT1 with AIS risk and also to explore its association with Th1 cells, Th17 cells, disease severity, and prognosis in AIS patients.

## 2 METHODS

### 2.1 Subjects

From January 2016 to December 2017, this study consecutively enrolled 120 first‐episode AIS patients. The inclusion criteria were as follows: (i) newly diagnosed as AIS in line with the American Stroke Association Guideline,^21^ (ii) age more than 18 years, (iii) admission to our hospital within 24 h after the symptom onset, and (iv) absent of intracranial hemorrhage. The exclusion criteria were (i) complicated with hematological system diseases or malignancies, (ii) complicated with immune system disease or active infection, (iii) had use of immunosuppressive agents within 6 months, (iv) had a history of stroke, and (v) breastfeeding or pregnant patients. Meanwhile, a total of 120 controls with age‐ and gender‐matched to AIS patients were enrolled in this study. The controls were subjects who underwent a physical examination in our hospital during the corresponding period. The eligible criteria for controls were (i) identified as high‐stroke risk population, which was defined as subjects with at least 2 of the following risk factors^22^: (a) hypertension, (b) atrial fibrillation or valvulopathy, (c) tobacco use, (d) hyperlipidemia, (e) diabetes, (f) lack of physical exercise, (g) overweight or obesity, and (h) family history of stroke; (ii) had no history of stroke or malignancies; (iii) without immune system disease or active infection; (iv) no use of immunosuppressive agents within 6 months; and (v) not in pregnancy or lactation. Moreover, in order to match the age and gender of the controls to the AIS patients, we limited the age of the controls to 45–80 years in the enrollment, and the sex ratio was set at 2:1 (male vs. female).

### 2.2 Ethics statement

All subjects or their family members had given the written informed consents before they were enrolled in the study. This study was conducted following the principles in the Helsinki Declaration. The Institutional Review Board approved the current study with approval number (2017‐001‐01).

### 2.3 Data and sample collection

Clinical characteristics of AIS patients and controls were documented after enrollment, which included age, gender, body mass index (BMI), current smoking status, and comorbidities. For AIS patients, the National Institutes of Health Stroke Scale (NIHSS) score was assessed on the day of admission for the assessment of disease severity. In addition, peripheral blood samples of AIS patients (on the day of admission) and controls were collected; meanwhile, the peripheral blood mononuclear cells (PBMCs) and the serum samples were collected by density gradient centrifugation. The CD4^+^ T cells were isolated from the PBCMs using a Dynabeads^®^ FlowComp™ Human CD4 kit (Invitrogen) following the protocol given by the manufacturer.

### 2.4 MALT1 detection by RT‐qPCR

For AIS patients and controls, MALT1 expression in the CD4^+^ T cells was determined by reverse transcription quantitative polymerase chain reaction (RT‐qPCR) assay. In brief, total RNA in the CD4^+^ T cells was separated by using TRIzol™ Reagent (Thermo Fisher Scientific). Then cDNA was synthesized using a ReverTra Ace^®^ qPCR RT Kit (Toyobo). After that, qPCR was conducted using SYBR^®^ Green Realtime PCR Master Mix (Toyobo). MALT1 relative expression was calculated by using the 2^−ΔΔC^ method using GADPH as internal reference. The sequence of primers used in the qPCR was listed as follows: MALT1, forward (5′‐>3′): AGTGTTGATGGCGTCTCTGAAT, reverse (5′‐>3′): TCTACCTTCTTGCTATCTTGACTGT; GAPDH, forward (5′‐>3′): TGACCACAGTCCATGCCATCAC, reverse (5′‐>3′): GCCTGCTTCACCACCTTCTTGA.

### 2.5 Th1 and Th17 cell proportion analysis

For AIS patients, Th1 and Th17 cells in the CD4^+^ T cells were determined by flow cytometric analysis with FlowJo10 software (BD Company, Franklin Lake, New Jersey, USA) using a Human Th1/Th17 Phenotyping Kit (BD Company). The identification of Th1 cell and Th17 cell was performed following the experiment protocol provided by the manufacturer. Then, Th1 and Th17 cell proportion in the CD4^+^ T cells was calculated.

### 2.6 Interferon‐gamma (IFN‐γ) and interleukin‐17 (IL‐17) determination

For AIS patients, IFN‐γ (known as Th1 main cytokine) and IL‐17 (known as Th17 main cytokine) in serum were assessed by enzyme‐linked immunosorbent assay (ELISA). Human ELISA kits of IFN‐γ and IL‐17 (Thermo Fisher Scientific) were applied for ELISA following the experiment protocol provided by the manufacturer.

### 2.7 Recurrence‐free survival evaluation

The surveillance and follow‐up of AIS patients after the occurrence of stroke were conducted in line with the AIS Guideline.^21^ All patients were consecutively followed up until stroke recurrence or death, or completion of a 36‐month scheduled follow‐up. The last follow‐up date was December 31, 2020. During the follow‐up, the date of stroke recurrence or death was documented for estimation of RFS.

### 2.8 Statistical analysis

Student's *t* test, chi‐square test, or Wilcoxon rank sum test was used for the difference analysis. Receiver operating characteristic (ROC) curve analysis was used to assess the value of variable in distinguishing subjects. The correlation analysis was completed by Spearman's rank correlation test. RFS was demonstrated by the Kaplan‐Meier curve and was determined by the log‐rank test between the two groups. Univariable and multivariable forward stepwise Cox's proportional hazard regression model analyses were carried out to evaluate the influence of variables on RFS. A *p* value <0.05 was considered statistical significant. Data analysis and graph plotting were completed by SPSS 24.0 statistical software (SPSS Inc) and GraphPad Prism 7.01 software (GraphPad Software Inc), respectively.

## 3 RESULTS

### 3.1 Study flow

**FIGURE 1:**
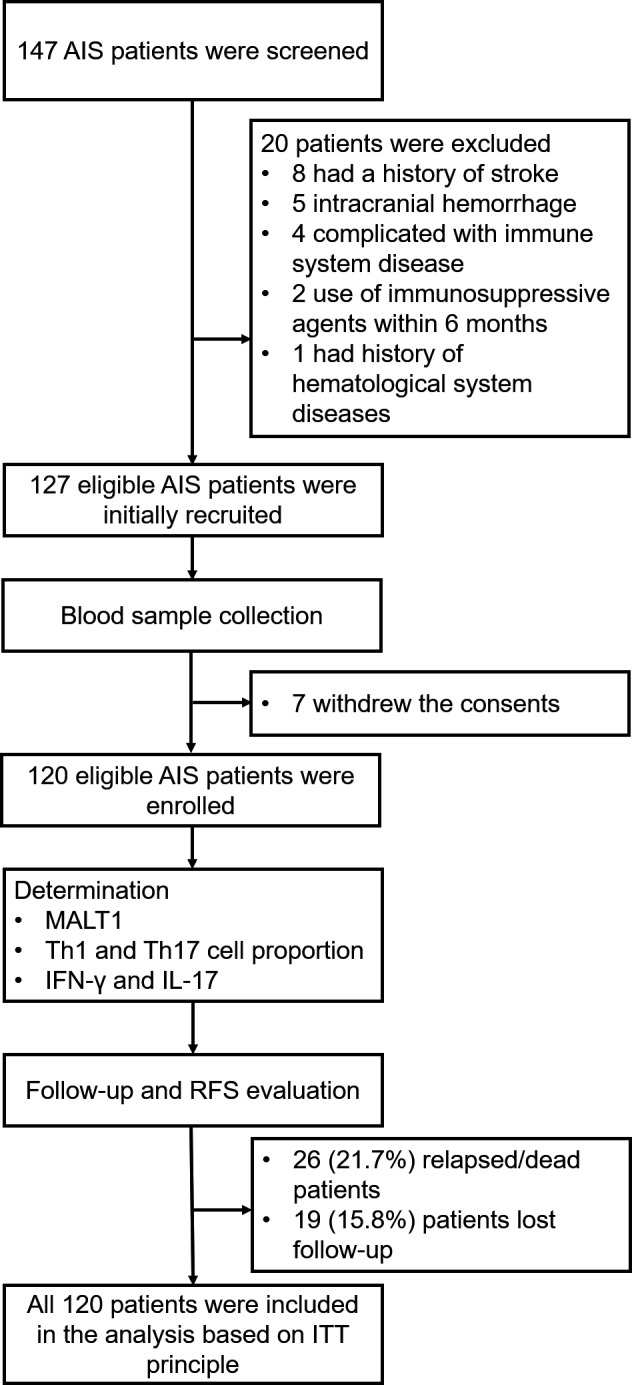
Study flow. AIS, acute ischemic stroke; IFN, interferon; IL, interleukin; ITT, intention to treat; MALT1, mucosa‐associated lymphoid tissue lymphoma translocation protein 1; RFS, recurrence‐free survival; Th1, T helper type 1; Th17, T helper type 17

Totally, 147 AIS patients were screened (Figure 1). Among which, 20 AIS patients were excluded including 8 patients who had a history of stroke, 5 patients who had intracranial hemorrhage, 4 patients who complicated with immune system disease, 2 patients who had history of immunosuppressive agents within 6 months, and 1 patient who had hematological system diseases. Then, 127 eligible AIS patients were initially recruited in this study, and their blood samples were collected. However, 7 patients withdrew their consents, leaving 120 AIS patients. In 120 AIS patients, their MALT1 expression, Th1 and Th17 cell proportion, IFN‐γ and IL‐17 levels were determined. During the follow‐up period, 26 patients (21.7%) experienced death or recurrence and 19 patients (15.8%) lost to follow‐up. All 120 AIS patients were included in the final analysis based on intention‐to‐treat (ITT) principle.

### 3.2 Patients’ clinical features

As listed in Table 1, the mean age of AIS patients and controls was 64.7 ± 9.4 years and 63.3 ± 9.1 years, respectively (Table 1). There were 45 (37.5%) female and 75 (62.5%) male AIS patients, then 40 (33.3%) female and 80 (66.7%) male subjects in controls. Moreover, the age (*p* = 0.240), gender (*p* = 0.500), BMI (*p* = 0.836), current smoking (*p* = 0.517), hypertension (*p* = 0.115), hyperlipidemia (*p* = 0.519), hyperuricemia (*p* = 0.058), diabetes mellitus (*p* = 0.150), and chronic kidney disease (*p* = 0.092) were different between AIS patients and controls. Furthermore, the mean value of the NIHSS score in AIS patients was 8.8 ± 3.6.

**TABLE 1:** Clinical features of AIS patients and controls

Items	Controls (*N* = 120)	AIS patients (*N* = 120)	Statistics (*χ* ^2^/*t*)	*p* Value
Age (years), mean ± SD	63.3 ± 9.1	64.7 ± 9.4	−1.179	0.240
Gender, No. (%)				
Female	40 (33.3)	45 (37.5)	0.455	0.500
Male	80 (66.7)	75 (62.5)		
BMI (Kg/m^2^), mean ± SD	24.1 ± 2.9	24.2 ± 2.4	−0.208	0.836
Current smoking, No. (%)				
No	68 (56.7)	63 (52.5)	0.420	0.517
Yes	52 (43.3)	57 (47.5)		
Hypertension, No. (%)				
No	24 (20.0)	15 (12.5)	2.480	0.115
Yes	96 (80.0)	105 (87.5)		
Hyperlipidemia, No. (%)				
No	63 (52.5)	58 (48.3)	0.417	0.519
Yes	57 (47.5)	62 (51.7)		
Hyperuricemia, No. (%)				
No	85 (70.8)	71 (59.2)	3.590	0.058
Yes	35 (29.2)	49 (40.8)		
Diabetes mellitus, No. (%)				
No	100 (83.3)	91 (75.8)	2.077	0.150
Yes	20 (16.7)	29 (24.2)		
CKD, No. (%)				
No	108 (90.0)	99 (82.5)	2.846	0.092
Yes	12 (10.0)	21 (17.5)		
NIHSS score, mean ± SD	—	8.8 ± 3.6	—	—

Abbreviations: AIS, acute ischemic stroke; BMI, body mass index; CKD, chronic kidney disease; NIHSS, National Institutes of Health Stroke Scale; SD, standard deviation.

### 3.3 Expressions of MALT1, IFN‐γ, and IL‐17

**FIGURE 2:**
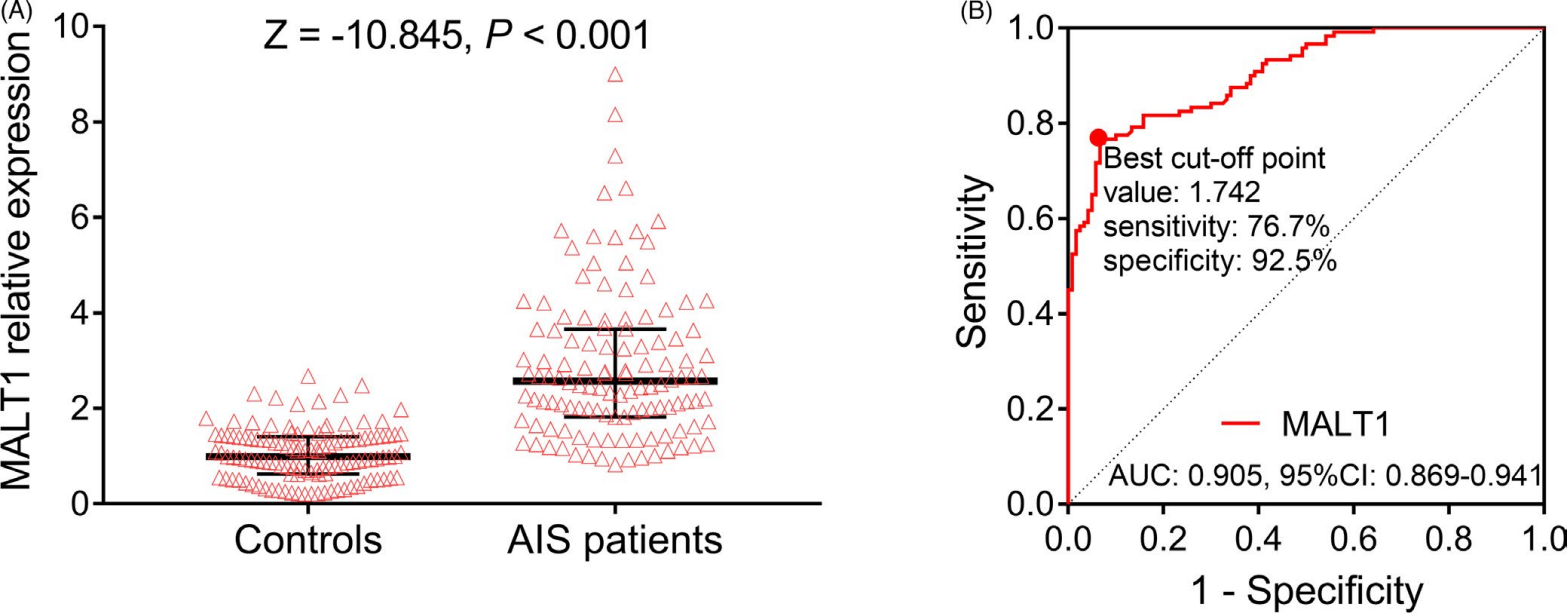
MALT1 expression was increased in AIS patients. MALT1 expression was increased in AIS patients compared with controls (A) and could distinguish AIS patients from controls (B). AIS, acute ischemic stroke; AUC, area under curve; CI, confidence interval; MALT1, mucosa‐associated lymphoid tissue lymphoma translocation protein 1; ROC, receiver operating characteristics

MALT1 expression was higher in AIS patients than controls (*p* < 0.001) (Figure 2A). Meanwhile, further ROC curve analysis revealed that MALT1 had a good ability in distinguishing AIS patients from controls, with an area under curve (AUC) of 0.905 (95% confidence interval [CI]: 0.869–0.941) (Figure 2B). Additionally, at the best cutoff point, the expression of MALT1 was 1.742, with specificity of 92.5% and sensitivity of 76.7%.

Besides, IFN‐γ (*p* < 0.001) (Figure S1A) and IL‐17 (*p* < 0.001) (Figure S1B) expressions were increased in AIS patients compared with controls as well.

### 3.4 Correlation of MALT1 with Th1 cells, Th17 cells, and their secreted cytokines

**FIGURE 3:**
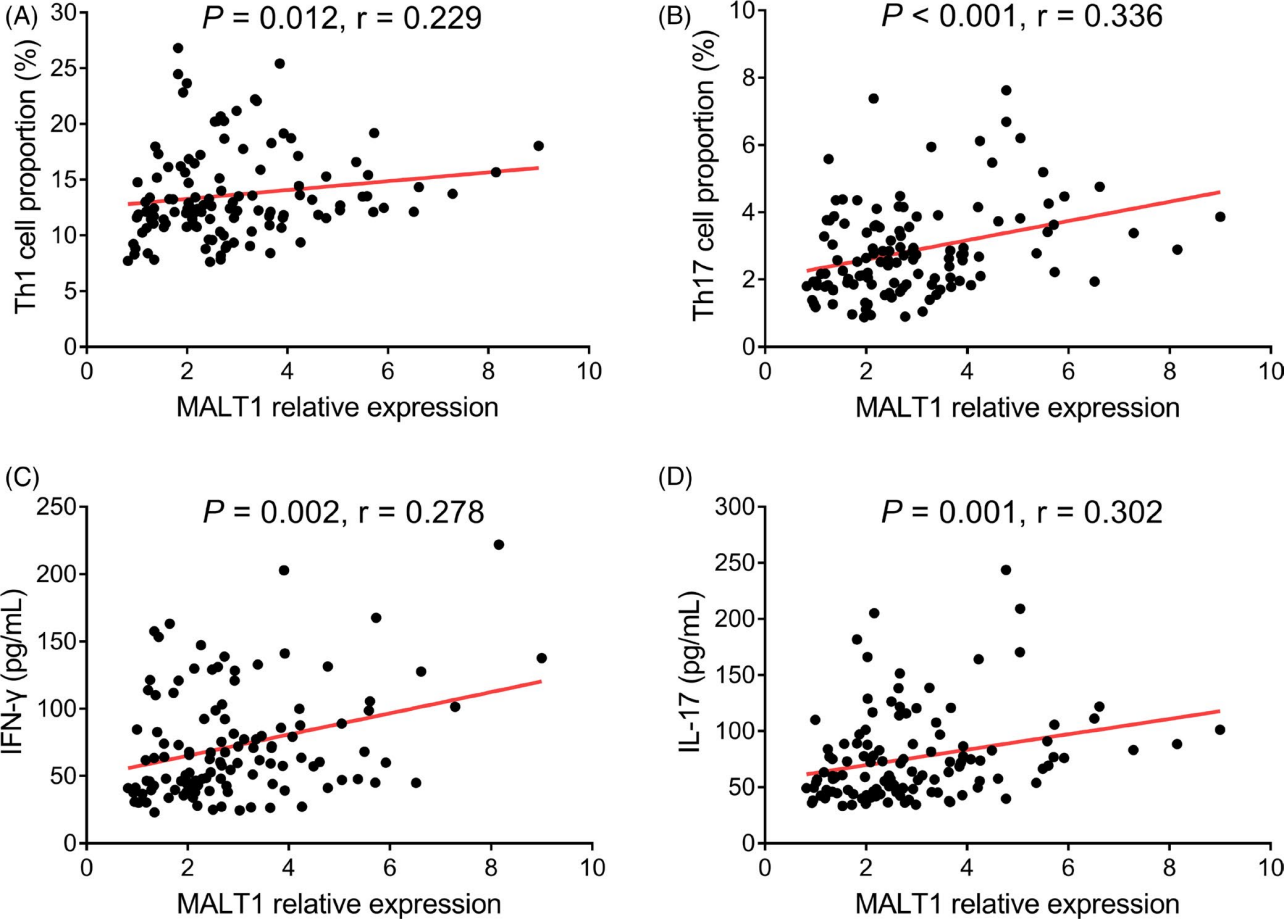
MALT1 positively correlated with Th1 and Th17 cells in AIS patients. MALT1 positively correlated with Th1 cells (A), Th17 cells (B), IFN‐γ (C), and IL‐17 (D). AIS, acute ischemic stroke; IFN, interferon; IL, interleukin; MALT1, mucosa‐associated lymphoid tissue lymphoma translocation protein 1; Th1, T helper type 1; Th17, T helper type 17

The detailed information regarding levels of Th1 cells, Th17 cells, and their secreted cytokines in AIS patients was shown in Table 2. Regarding their correlation, MALT1 positively correlated with Th1 cells (*r* = 0.229, *p* = 0.012, Figure 3A), Th17 cells (*r* = 0.336, *p* < 0.001, Figure 3B), IFN‐γ (*r* = 0.278, *p* = 0.002, Figure 3C), and IL‐17 (*r* = 0.302, *p* = 0.001, Figure 3D).

**TABLE 2:** The level of Th1 cell, Th17 cell, IFN‐γ, and IL‐17 in the AIS patients

Items	AIS patients (*N* = 120)
Th1 cell ratio (%)	
Mean ± SD	13.6 ± 4.1
Median (IQR)	12.4 (11.1 ~ 15.7)
Range	7.6 ~ 26.8
Th17 cell ratio (%)	
Mean ± SD	2.9 ± 1.4
Median (IQR)	2.6 (1.9 ~ 3.6)
Range	0.9 ~ 7.6
IFN‐γ (pg/ml)	
Mean ± SD	72.2 ± 40.3
Median (IQR)	60.3 (42.7 ~ 92.3)
Range	23.0 ~ 221.9
IL−17 (pg/ml)	
Mean ± SD	75.9 ± 40.0
Median (IQR)	63.3 (46.7 ~ 88.5)
Range	33.4 ~ 243.6

Abbreviations: AIS, acute ischemic stroke; IFN‐γ, interferon‐gamma; IL‐17, interleukin‐17; IQR, interquartile range; SD, standard deviation; Th1, T helper type 1; Th17, T helper type 17.

### 3.5 Correlation of MALT1 with NIHSS score

**FIGURE 4:**
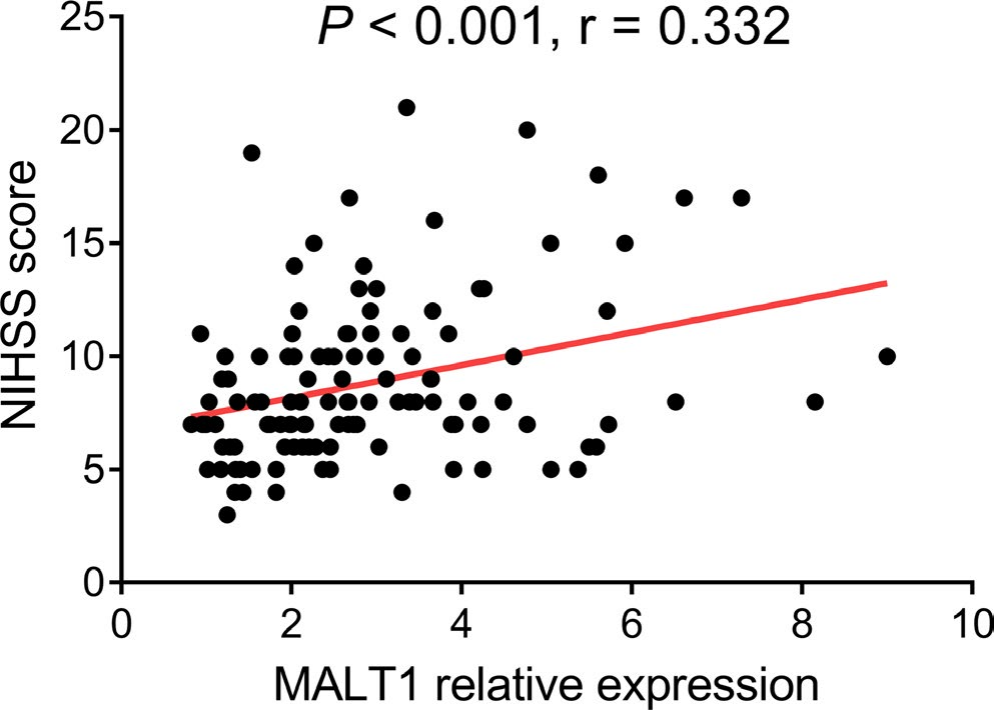
MALT1 positively correlated with the NIHSS score in AIS patients. AIS, acute ischemic stroke; MALT1, mucosa‐associated lymphoid tissue lymphoma translocation protein 1; NIHSS, National Institutes of Health Stroke Scale

To investigate the association of MALT1 with disease severity featured by the NIHSS score in AIS patients, correlation analysis was conducted and discovered that MALT1 positively associated with the NIHSS score (*r* = 0.332, *p* < 0.001) in AIS patients (Figure 4).

### 3.6 Correlation of MALT1, IFN‐γ, and IL‐17 with RFS

**FIGURE 5:**
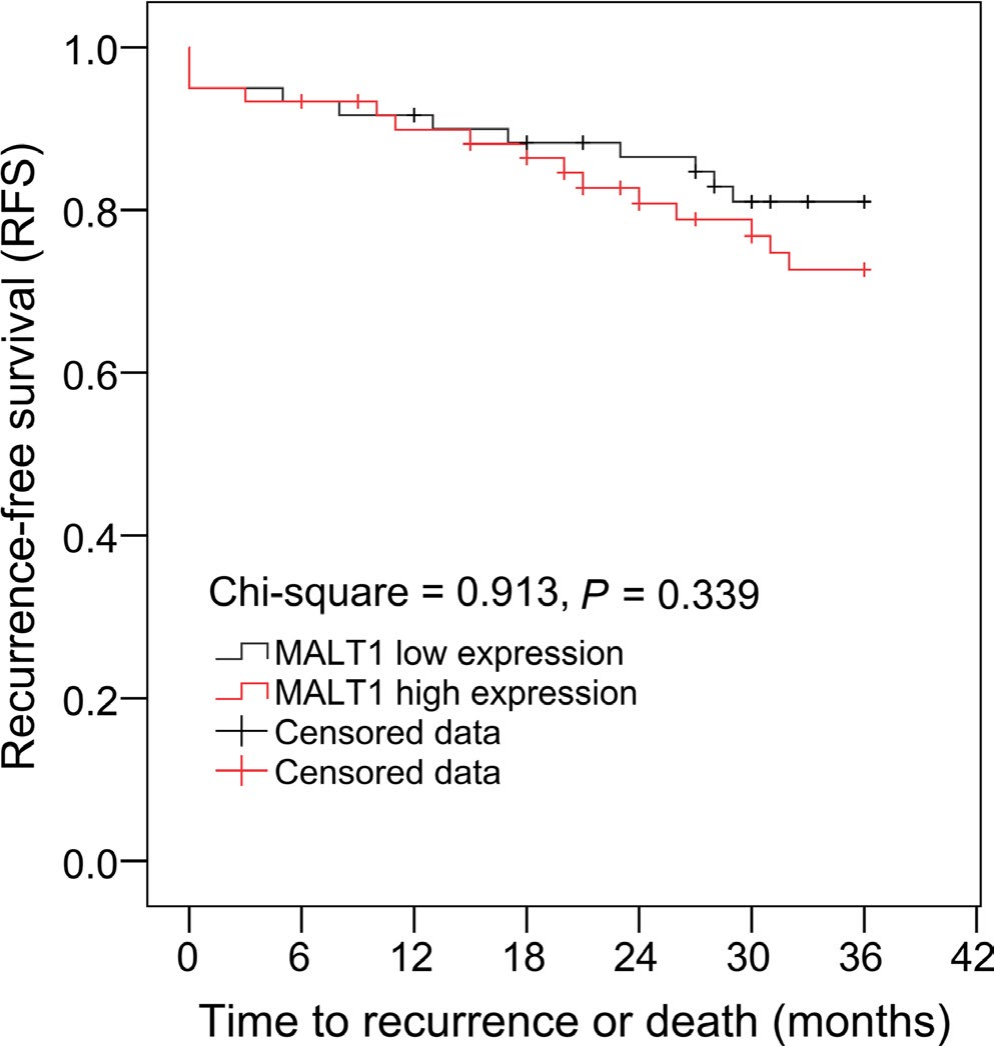
AIS patients with high MALT1 expression and AIS patients with low MALT1 expression exhibited similar RFS. AIS, acute ischemic stroke; MALT1, mucosa‐associated lymphoid tissue lymphoma translocation protein 1; RFS, recurrence‐free survival

**FIGURE 6:**
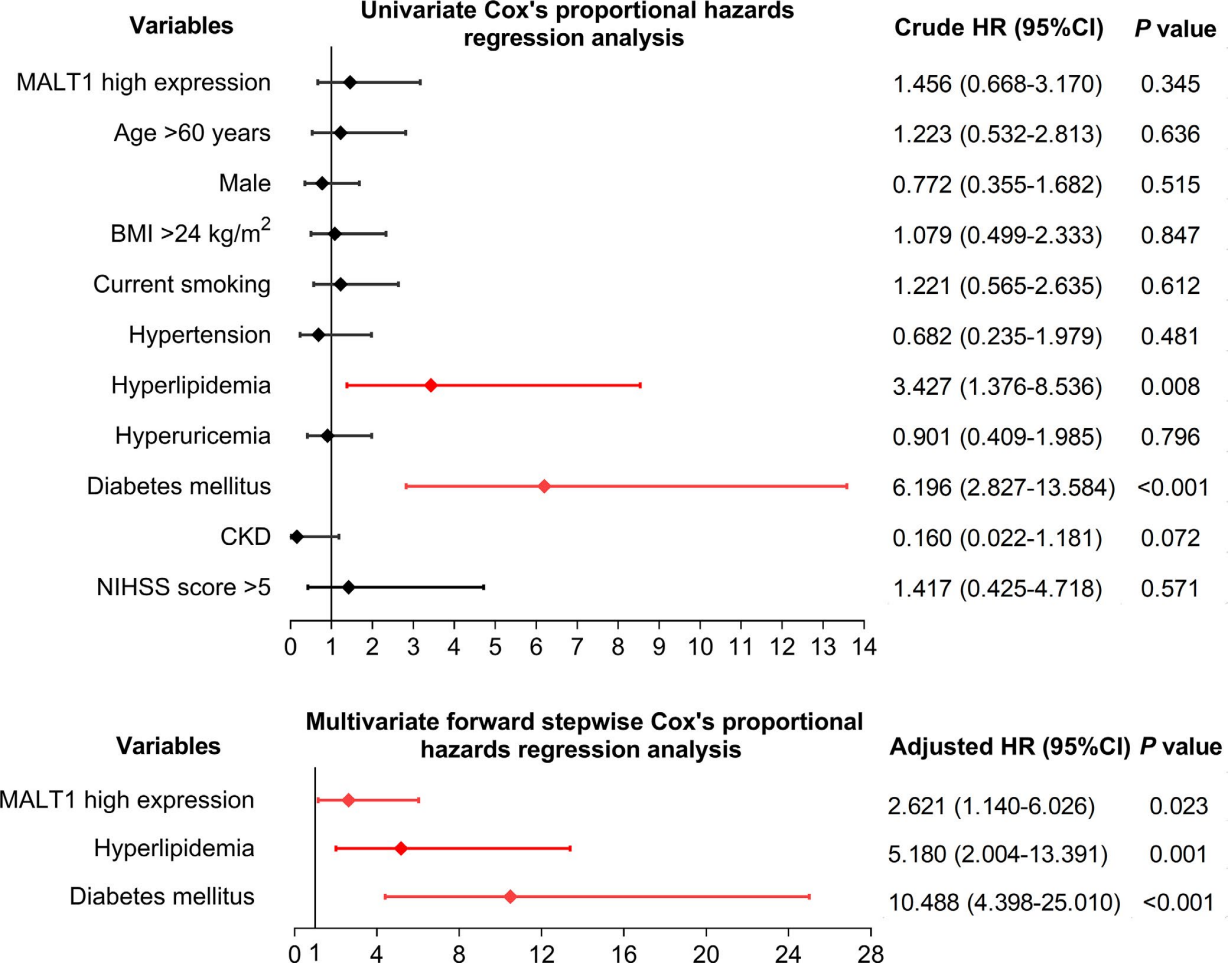
MALT1, hyperlipidemia and diabetes mellitus independently correlated with reduced RFS in AIS patients. AIS, acute ischemic stroke; BMI, body mass index; CKD, chronic kidney disease; HR, hazard ratio; MALT1, mucosa‐associated lymphoid tissue lymphoma translocation protein 1; RFS, recurrence‐free survival

There was no correlation of MALT1 expression with RFS in AIS patients by observing the K‐M curve (*p* = 0.339) (Figure 5). Further univariate Cox's regression analysis also showed no correlation of high MALT1 expression with RFS in AIS patients (*p* = 0.345) (Figure 6). To eliminate the confounding factors, multivariant Cox's regression analysis was performed, which showed that high MALT1 expression independently associated with shorter RFS in AIS patients (hazard ratio [HR]: 2.621, 95% CI: 1.140–6.026, *p* = 0.023).

Besides, high IL‐17 expression was correlated with shorter RFS (*p* = 0.022) (Figure S2B), and there was no correlation of IFN‐γ expression with RFS (*p* = 0.067) (Figure S2A) in AIS patients.

## 4 DISCUSSION

MALT1, a paracaspase which refers to caspase homolog, displays highly structural similarity as caspase involving in pro‐inflammatory cytokine regulation.^7^ For example, MALT1 promotes inflammatory cytokine production, CD4^+^ T‐cell differentiation, and T‐cell activation by participating in the NF‐κB signaling pathway in myeloid cells.^8^ Moreover, MALT1 also induces vascular inflammation by activating the NF‐κB signaling pathway in nonmyeloid cells (such as endothelial cells).^14^, ^15^, ^16^, ^17^ Although MALT1 promotes vascular inflammation and induces endothelial dysfunction, no relevant study investigates its clinical application in AIS patients. Thus, we conducted this study and discovered that MALT1 expression was elevated in AIS patients compared with controls, it could also distinguish AIS patients from controls. This might be explained as follows: (a) MALT1 might regulate cylindromatosis (CYLD) cleavage, thereby leading to microtubule dysfunction and endothelial cell barrier disruption, thus further resulted in higher paracellular permeability and leakage into the subendothelial space, therefore eventually resulted in the elevation of AIS risk.^14^ (b) MALT1 might promote the production of pro‐inflammatory cytokines and further led to vascular inflammation by the formation of caspase recruitment domain family member 10 (CARMA3)–(BCL10 immune signaling adaptor) Bcl10‐MALT1 signalosome in the endothelium, thereby resulted in elevated AIS risk.^15^

Previous studies indicate that MALT1 regulates CD4^+^ T‐cell differentiation into Th1 and 17 cells, and their effector response based on in vitro study.^12^ Meanwhile, in vivo, the MALT1 deficiency colitis mouse model displayed a reduction of the Th17 cell amount and IL‐17A (Th17 cell secreted cytokines).^11^ Furthermore, MALT1 deficiency resulted in a declined T‐cell proliferation as well as unbalanced regulatory T‐cell and effector T‐cell amount, which further led to inflammation of multiorgan.^13^ However, no relevant clinical study explores the correlation of MALT1 with Th1 and Th17 cells in cerebrovascular disease patients such as AIS patients. Therefore, in this study, we found that MALT1 positively correlated with Th1 cells, Th17 cells, and their secreted cytokines in AIS patients. The possible reason might be due to its proteolytic activity, abnormal expression of MALT1 might be involved in the elicitation of more proteins, which involved in T‐cell differentiation and proliferation, thus further leading to the impairment of Th1 cells, Th17 cells, and their secreted cytokines in AIS patients. In addition, we also discovered that MALT1 positively correlated with disease severity of AIS patients, which might be explained as that MALT1 might cause an increased production of pro‐inflammatory cytokines by regulating the NF‐κB signaling pathway, as mentioned earlier, thus further leading to more advanced disease severity in AIS patients.^7^, ^8^, ^9^

In the present study, we also investigated the correlation of MLAT1 expression with prognosis in AIS patients, and it showed that high MALT1 expression was independently correlated with shorter RFS. The possible reasons were as follows: (a) as mentioned earlier, higher MALT1 expression could promote vascular inflammation and endothelial dysfunction, thereby leading to an unfavorable prognosis in AIS patients.^14^, ^15^, ^16^, ^17^ (b) MALT1 positively correlated with disease severity in AIS patients, as discussed earlier. Therefore, more advanced disease severity might result in shorter RFS in AIS patients.

Several clinical utilities of our findings were discovered: (a) MLAT1 and Th17 cell measurement might provide additional assistance for AIS diagnosis and prognostication; (b) the interaction between MALT1 and Th1/Th17 cell differentiation might be involved in AIS pathogenesis, although further exploration was needed. However, there were some limitations in the present study. First, our sample size was relatively small; thus, a study with a larger sample size to validate the correlation of MALT1 with inflammation and disease severity in AIS patients was needed. Second, further study would detect IFN‐γ and IL‐17 expressions in AIS patients over time to explore their longitudinal change in AIS patients and their correlation with treatment efficacy. Finally, the detailed interacting mechanism underlying MALT1 with Th1 and Th17 cells was not explored in the present study, which could be investigated in the further study.

In conclusion, MALT1 expression is increased and positively correlates with disease severity, Th1 cells, and Th17 cells, whose high expression severs as an independent risk factor for shorter RFS in AIS patients.

## CONFLICT OF INTEREST

The authors declare that they have no conflicts of interest.

## Supporting information

Fig S1

Fig S2

## Data Availability

Data sharing not applicable to this article as no datasets were generated or analyzed during the current study.
