# Supplementary figures and images for: MALT1 positively correlates with Th1 cells, Th17 cells, and their secreted cytokines and also relates to disease risk, severity, and prognosis of acute ischemic stroke
Source: J Clin Lab Anal. 2021 Jul 17;35(9):e23903. doi: 10.1002/jcla.23903 (PMC8418463; doi:10.1002/jcla.23903)

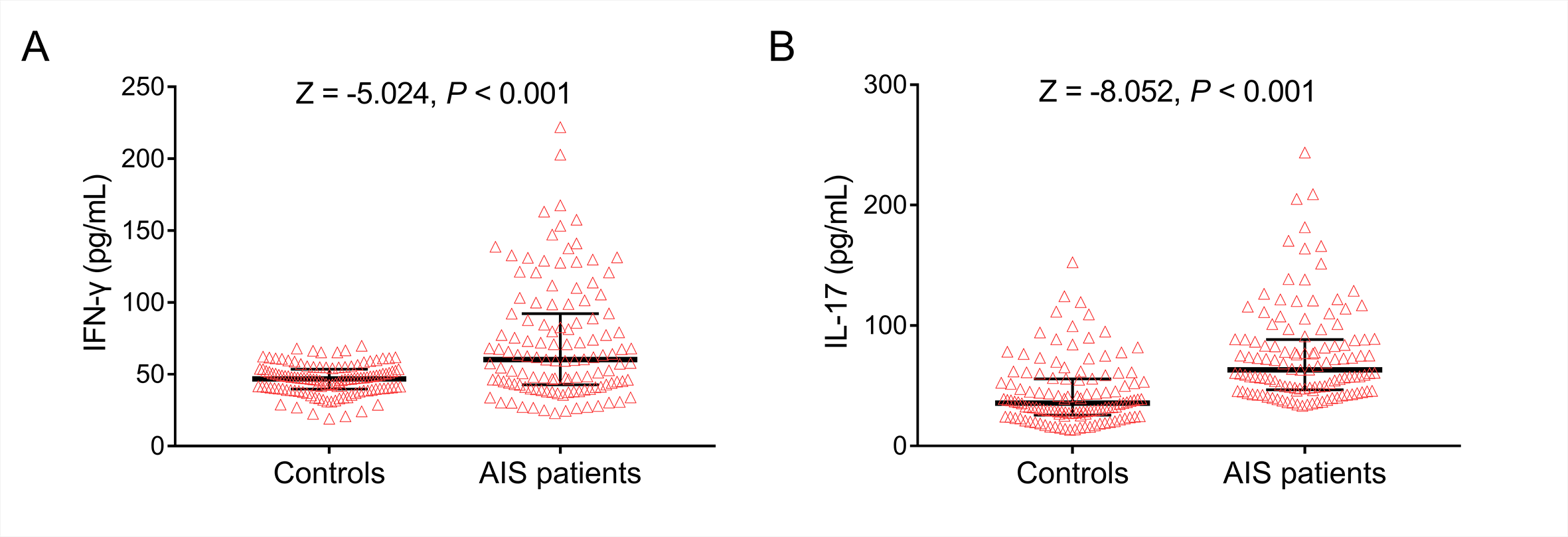

Supplement: Supplementary file 1 — Fig S1 [file JCLA-35-e23903-s002.tif]

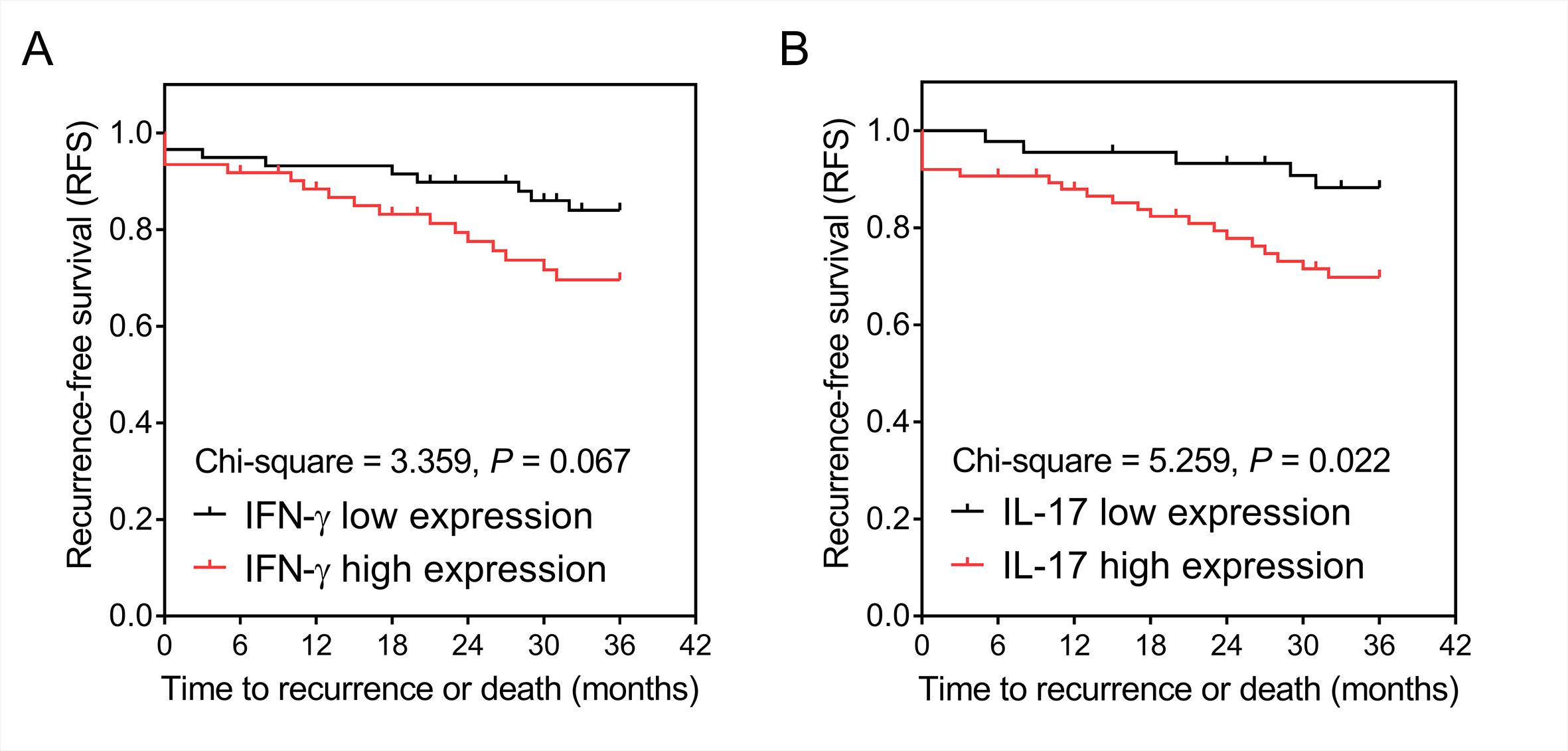

Supplement: Supplementary file 2 — Fig S2 [file JCLA-35-e23903-s001.tif]
